# Association of dietary and lifestyle inflammation score (DLIS) with chronic migraine in women: a cross-sectional study

**DOI:** 10.1038/s41598-024-66776-6

**Published:** 2024-07-16

**Authors:** Farnush Bakhshimoghaddam, Davood Shalilahmadi, Reza Mahdavi, Zeinab Nikniaz, Majid Karandish, Samaneh Hajjarzadeh

**Affiliations:** 1 Student Research Committee, Ahvaz Jundishapur University of Medical Sciences, Ahvaz, Iran; 2 Nutrition and Metabolic Diseases Research Center, Clinical Sciences Research Institute, Ahvaz Jundishapur University of Medical Sciences, Ahvaz, Iran; 3 Department of Nutrition, School of Allied Medical Sciences, Ahvaz Jundishapur University of Medical Sciences, Ahvaz, Iran; 4 School of Medicine, Ahvaz Jundishapur University of Medical Sciences, Ahvaz, Iran; 5 Nutrition Research Center, Tabriz University of Medical Sciences, Tabriz, Iran; 6 Liver and Gastrointestinal Diseases Research Center, Tabriz University of Medical Sciences, Tabriz, Iran

**Keywords:** Migraine, Diet, Lifestyle, Inflammation, DLIS, Women, Headache, Neuroscience, Diseases

## Abstract

Due to more frequent and intense attacks, chronic migraine (CM) sufferers usually report more disability compared to patients with episodic migraine (EM). There is increasing evidence that points to inflammatory diet and lifestyle as a probable underlying cause of migraine. The present study investigated the association of dietary and lifestyle inflammation scores (DLIS) with the odds of CM in Iranian women. In the current study, 285 women with migraine enrolled. Migraine was diagnosed by a single neurologist based on the third edition of the International Classification of Headache Disorders (ICHD-III). The women were categorized into CM and EM groups based on their attack frequency per month. Adherence to the dietary inflammation score (DIS), Lifestyle Inflammatory Score (LIS), and DLIS (DIS + LIS) was assessed based on last year’s dietary intakes collected using a semi-quantitative food frequency questionnaire (FFQ). The Odds Ratio (OR) for CM across the DIS, LIS, and DLIS tertiles were assessed through logistic regression. Most of the participants were overweight or obese (74.4%). The percentage of women with CM was 40.7%. Women with CM had significantly higher DIS (*P* = 0.002) and DLIS (*P* = 0.04) than women with EM. There was a significant positive association between CM and DIS. Those in the third tertile of the DIS had almost two times higher chance of experiencing chronic migraine compared with those in the first tertile [OR = 2.02; 95% CI 1.06–3.82; *P* = 0.03]. the *P*-value for the trend also was significant (0.03). In terms of LIS and DLIS tertiles, no significant association was observed. Adherence to the more inflammatory diets was associated with higher chances of experiencing CM in women.

## Introduction

Migraine is a neurological disorder characterized by recurrent unilateral or bilateral headache attacks, typically pulsating in quality and ranging in intensity from moderate to severe, depending on the lifestyle and genetic background^1^. It is estimated that 15% of the global population suffers from migraines, with women being two to three times more likely to be affected than men^2^. Migraine can be classified into chronic and episodic subtypes based on the frequency of attacks. Chronic migraine sufferers usually report more frequent and intense migraine headaches and thus more migraine-related disability compared to patients with episodic migraine. Lack of control and ineffective treatment of episodic migraine can cause the progression of chronic migraine in the long term period^3^.

The pathophysiology of migraine remains poorly understood. Although inflammation in the vascular system, activation of the neurogenic and trigeminovascular systems, corticotropin-releasing hormone, and changes in the immunologic system have been implicated in the etiology of migraine^4,5^. It has been demonstrated that oxidative stress and systemic inflammation play a significant role in migraine headaches^6^. Previous research has found that migraine patients have significantly higher concentrations of inflammatory factors such as tumor necrosis factor-α (TNFα), C-reactive protein (CRP), and interleukins^7^.

Many studies have been conducted over the past few years aiming to reduce the risk of migraine through dietary and lifestyle modification. It is accepted that diets rich in fruits, vegetables, nuts, legumes, and whole grains, can reduce systemic inflammation effectively. In contrast, diets high in saturated and trans fats, refined grains, and simple sugars can induce the overproduction of pro-inflammatory cytokines, leading to the activation of the innate immune system^8^. In addition, lifestyle factors such as abdominal obesity^9^, physical inactivity^10^, smoking^11^, and alcohol consumption^12^ contribute to low-grade systemic inflammation. Recently, Byrd et al.^13^ introduced novel inflammatory indices, including dietary inflammation scores (DIS) and lifestyle inflammation scores (LIS), based on the association between food groups and lifestyle behaviors and the status of inflammatory markers. Focusing on DIS and LIS may provide a more accurate view of the relationship between human lifestyle as a whole and migraine disorder development than that of a single component such as obesity or diet.

To our knowledge, no study has yet examined the association between DIS and LIS and migraine disease. Previous studies have investigated another diet-related inflammation score, such as the diet-related inflammation index (DII), and its association with migraine characteristics^14–16^. However, the DII has some limitations and does not indicate lifestyle parameters^17^. Therefore, the current study was designed to investigate the associations between DIS, LIS, and the dietary and lifestyle inflammation score (DLIS) and the odds of chronic migraine in Iranian women.

## Materials and method

### Study design and participants

This study is a secondary analysis of our previous data collection to assess dietary patterns and quality in 300 women with migraine^18,19^. The inclusion criteria were women aged 25–55 years who were referred to the neurology clinics of Golestan Hospital in Ahwaz, Iran, from July 2017 to March 2018. Exclusion criteria were women who had a history of asthma, epilepsy, thyroid, and hormonal disorders, and who were pregnant or lactating. The research was conducted in accordance with the Declaration of Helsinki. All patients read and signed the written consent form. The study protocol was approved by the Ethics Committee of Tabriz University of Medical Sciences (IR.TBZMED.REC.1395.1285) on 28th October 2017 and Ahvaz Jundishapur University of Medical Sciences (IR.AJUMS.REC.1396.705) on 20th February 2017.

### Migraine diagnosis

Migraine diagnosis and aura status were determined by a single neurologist using the third edition of the International Classification of Headache Disorders (ICHD-III) criteria^20^ for migraine. A visual analog scale (VAS) was used as a criterion for pain intensity. Women reported their migraine attack frequency as the number of attacks per month. Participants who had 15 or more attacks per month were classified as having chronic migraine, otherwise, they were classified as having episodic migraine^20^.

### Demographic characteristics and anthropometric measurements

The demographic and anthropometric assessments of the participants were collected via face-to-face interviews. At the first neurology clinic visit, trained interviewers obtained information on age, marital status (married or unmarried), education (less than high school, high school graduate, and college graduate), occupation (employed or unemployed), and smoking status (never smoker or current smoker) using predesigned data extraction forms. The body weight and height of study participants were measured by a single expert to eliminate measurement errors. Body mass index (BMI) (kg/m^2^) was calculated by dividing weight (kg) by height (m^2^).

### Physical activity and dietary assessment

Physical activity was assessed using the short form of the International Physical Activity Questionnaire (IPAQ) and reported as MET (metabolic equivalent min/week)^21^. The information from the MET was categorized into three levels (low, moderate, and high levels of physical activity). Participants’ usual dietary intake over the past year was collected using a reliable and validated semi-quantitative food frequency questionnaire (FFQ)^22,23^. Participants reported the frequency of consumption of 147 questionnaire foods on daily, weekly, monthly, and annual scales. All data were converted into daily intakes. Household measures were used to estimate grams of portion sizes consumed daily^24^. The total daily intakes of energy, protein, carbohydrate, fiber, and fat for each participant were calculated using the Nutritionist IV software based on the grams of food items on the questionnaire.

### Calculation of DIS, LIS, and DLIS

The FFQ-based DIS and the lifestyle questionnaire-based LIS for each participant were calculated using the presented Byrd et al.^10^ methods. The DIS consists of 19 components, including 18 whole foods and beverages and one composite micronutrient supplement group, which may affect the levels of anti-inflammatory biomarkers such as interleukin-10 (IL-10), as well as pro-inflammatory biomarkers such as CRP, interleukin-6 (IL-6), and IL-8. The inflammatory potential of each component was scored according to its ability to decrease or increase the levels of pro- and anti-inflammatory markers. The DIS includes leafy greens and cruciferous vegetables, tomatoes, apples, and berries, deep yellow or orange vegetables and fruits, other fruits, and real fruit juices, other vegetables, legumes, fish, poultry, red and organ meats, processed meats, added sugars, high-fat dairy, low-fat dairy, coffee and tea, nuts, other fats, refined grains, starchy vegetables, and the supplement score. Since we lack data on dietary supplement intake, we used 18 food groups to calculate the overall score. Dietary components were considered continuous variables (g/d), standardized to a mean of 0 and a standard deviation of 1.0., multiplied by the weight (β coefficient), and then summed (Supplemental Table 1).

The LIS included four inflammation-related lifestyle components: BMI, physical activity, smoking status, and alcohol consumption. Since Iranians generally do not consume alcohol or report it due to religious beliefs and legal restrictions, alcohol consumption was excluded from the LIS calculation. Individuals were categorized as average weight (BMI < 25), overweight (25 BMI < 30), and obese (BMI 30); and then received 0.0, 0.89, and 1.57 scores, respectively. The physical activity was divided into tertiles and the participants in the first, second, and third tertiles were given a score of 0.0, 0.19, and 0.41, respectively. Additionally, 0.50 and 0.0 were assigned as the suggested regression coefficients for smokers and non-smokers, respectively. To determine the LIS, we summed all the weighted values together. The scores of DIS and LIS were also added to calculate DLIS. Higher DIS, LIS, and DLIS (more positive) indicate an inflammatory diet and lifestyle, while lower DIS, LIS, and DLIS (more negative) indicate the opposite.

### Statistical analysis

SPSS software (SPSS Inc., Chicago, IL, version 24) was used to assess the associations. Anthropometric, demographic, and migraine characteristics of participants were categorized by tertiles of DLIS. The significance of the difference between tertiles was assessed by the Kruskal-valise test for unnormal distributed quantitative variables and Chi-square tests (linear by linear and Fisher’s exact test) for qualitative variables. DIS, LIS, and DLIS components of women with episodic and chronic migraine were compared using the Mann–Whitney test and Fisher’s exact test wherever it was appropriate. The Odds Ratio (95% Confidence Interval) for chronic migraine across the CM across DIS, LIS, and DLIS tertiles was determined using logistic regression analysis in crude and adjusted models (age, marital status, occupation status, education level, migraine type, family history, pain intensity, pain duration, and energy intake in case of LIS and DLIS and adjusted for age, marital status, occupation status, education level, migraine type, family history, pain intensity, pain duration, energy intake, BMI category, smoking, and physical activity level in case of DIS).

## Result

Anthropometric and demographic characteristics of participants categorized by DLIS tertiles are displayed in Table 1. Most of the women in the study were overweight (40.7%) or obese (33.7%). There were significant differences in the level of education and occupational status of the recruited women in the different tertiles of the DLIS (*P* < 0.001).

**Table 1:** Anthropometric and demographic characteristics of participants categorized by DLIS score tertiles.

Characteristics	Total N = 285	Tertile 1 N = 95	Tertile 2 N = 95	Tertile 3 N = 95	*P*-value
Age (year)^*&*^	35 (15)	36 (15)^π,ɣ^	34 (15)^π,β^	34 (15)^ɣ,β^	0.73^a^
Energy intake (kcal/day)^*&*^	2945.80 (1424.06)	2691.14 (1023.59)^∆^	2697.68 (1203.57)^€^	3666.56 ± 1063.48^∆,€^	< 0.001^a^
Body mass index (Kg/M^2^)^*&*^	27.98 (6.14)	26.08 ± 4.35	27.52 ± 4.97	30.38 (7.45)	< 0.001^a^
Body mass index category					
Underweight (BMI < 18.5)	6 (2.1)	1 (1.1)	4 (4.2)	1 (1.1)	< 0.001^b^
Normal (18.5 ≤ BMI < 25)	68 (23.9)	42 (44.2)	22 (23.2)	4 (4.2)	
Overweight (25 ≤ BMI < 30)	116 (40.7)	38 (40)	43 (45.3)	35 (36.8)	
Obese (BMI ≥ 30)	95 (33.3)	14 (14.7)	26 (27.4)	55 (57.9)	
Marital status					
Not Married	65 (22.9)	29 (30.5)	19 (20)	17 (17.9)	0.28^c^
Married	220 (77.1)	66 (69.5)	76 (80)	78 (82.1)	
Education level					
Lower than high school	156 (54.7)	42 (44.2)	48 (50.5)	66 (69.5)	< 0.001^c^
High school graduated	62 (21.8)	19 (20)	24 (25.3)	19 (20)	
Higher than high school	67 (23.5)	34 (35.8)	23 (24.2)	10 (10.5)	
Occupation status					
Employed	59 (20.7)	28 (29.5)	23 (24.2)	8 (8.4)	< 0.001^c^
Non employed	226 (79.3)	67 (70.5)	72 (75.8)	87 (91.6)	
Smoking					
No	282 (98.9)	95 (100)	93 (97.9)	94 (98.9)	0.55^c^
Yes	3 (1.1)	0	2 (2.1)	1 (1.1)	
Physical activity^#^ (Met)					
Low	58 (69.1)	58 (61.7)	60 (63.2)	79 (83.2)	< 0.001^c^
Moderately physically active	70 (24.6)	28 (29.5)	26 (27.4)	16 (16.8)	
Heavily physically active	18 (6.3)	9 (9.5)	9 (9.5)	0	

^&^Unnormal and normal distributed variables were reported based on the median (interquartile range) and mean ± standard deviation, respectively. Qualitative variables were reported as frequency (percentage). DLIS: dietary and lifestyle inflammation score.

^a^*P*-value based on the Kruskal–valise test. Significant differences were assessed based on pairwise comparisons between tertiles using the Mann–Whitney test.

^b^*P*-value based on the Linear by Linear. ^c^*P*-value based on the Fisher’s Exact test.

^#^Physical activity based on Met (metabolic equivalent min/week).

Table 2 shows the migraine characteristics of participants categorized by DLIS tertiles. About 41% of women reported a migraine family history. In terms of the frequency of migraine attacks, 59.3% and 40.7% of women were classified in the EM and CM groups, respectively. Most of the participants experienced moderate (4–7 VAS score) and severe (8–10 VAS score) migraine attacks (44.2% and 55.4% respectively).

**Table 2:** Migraine characteristics of participants categorized by DLIS score tertiles.

Characteristics	Total N = 285	Tertile 1 N = 95	Tertile 2 N = 95	Tertile 3 N = 95	*P*-value
Migraine type					
With aura	26 (9.1)	86 (90.5)	86 (90.5)	87 (91.6)	1^a^
Without aura	259 (90.9)	9 (9.5)	9 (9.5)	8 (8.4)	
Family history					
Yes	116 (40.7)	39 (41.1)	34 (35.8)	43 (45.3)	0.07^a^
No	169 (59.3)	56 (58.9)	61 (64.2)	52 (54.7)	
Pain duration					
< 4 h	111 (38.9)	36 (37.9)	31 (32.6)	44 (46.3)	0.51^a^
4–24 h	98 (34.4)	36 (37.9)	35 (36.8)	27 (28.4)	
25–48 h	32 (11.2)	10 (10.5)	12 (12.6)	10 (10.5)	
49–72 h	13 (4.6)	4 (4.2)	7 (7.4)	2 (2.1)	
> 72 h	31 (10.9)	9 (9.5)	10 (10.5)	12 (12.6)	
Attack frequency					
Episodic migraine (< 15 attacks/month)	169 (59.3)	57 (60)	63 (66.3)	49 (51.6)	0.12^a^
Chronic migraine (≥ 15 attacks/month)	116 (40.7)	38 (40)	32 (33.7)	46 (48.4)	
Pain intensity (VAS)^&^					
1–3	1 (0.4)	0	0	1 (1.1)	0.12^a^
4–7	126 (44.2)	50 (52.6)	40 (42.1)	36 (37.9)	
8–10	158 (55.4)	45 (47.4)	55 (57.9)	58 (61.1)	

Qualitative variables were reported as frequency (percentage). DLIS: dietary and lifestyle inflammation score.

^&^Pain intensity based on visual analog scale (VAS).

^a^*P*-value based on the Fisher’s Exact test.

Table 3 shows a comparison of the DIS, LIS, and DLIS components between women with episodic and chronic migraine. In terms of DIS components, the women with EM had significantly higher consumption of “leafy greens and cruciferous vegetables” (*P* = 0.002), “apples and berries” (*P* = 0.006), “deep yellow or orange vegetables and fruit” (*P* = 0.004) and “other fruits and real fruit juices” (*P* = 0.002) than women with CM. In contrast, the women with CM reported a higher intake of “other fat” (*P* = 0.02) than women with EM. Moreover, the total DIS was significantly higher in women with CM than in women with EM (*P* = 0.002). There was no significant difference in the LIS components between the two groups. The DLIS (the sum of two previous scores) was significantly higher in women with CM than in women with EM (*P* = 0.04).

**Table 3:** Comparison of DIS, LIS, and DLIS scores components between women with EM and CM.

Indices	weight	Total N = 285	Episodic migraine 169 (59.3%)	Chronic migraine 116 (40.7%)	*P*-value^a^
DIS score					
Leafy greens and cruciferous vegetables	− 0.14	36.68 (43.92)	40.86 (47.15)	29.23 (39.78)	**0.002**
Tomatoes	− 0.78	83.54 (83.54)	83.54 (83.54)	82.17 (96.01)	0.49
Apples and berries	− 0.65	28.75 (40.40)	29.62 (39.03)	27.31 (35.51)	**0.006**
Deep yellow or orange vegetables and fruit	− 0.57	53.32 (55.91)	64.88 (58.74)	46.58 (51.10)	**0.004**
Other fruits and real fruit juices	− 0.16	276.62 (229.55)	303.07 (210.39)	230.64 (210.94)	**0.002**
Other vegetables	− 0.16	129.56 (111.59)	133.17 (96.74)	121.13 (136.39)	0.34
Legumes	− 0.04	46.61 (48.21)	47.77 (49.32)	46.41 (41.88)	0.40
Fish	− 0.08	15.34 (33.74)	16.12 (43.49)	14.56 (36.13)	0.27
Poultry	− 0.45	47.58 (35.42)	47.58 (29.80)	47.37 (41.42)	0.48
Red and organ meats	0.02	19.61 (18.99)	20.26 (19.94)	19.36 (18.17)	0.60
Processed meats	0.68	0.88 (3.99)	0.66 (3.01)	1.07 (5.33)	0.12
Added sugars	0.56	57.87 (62.06)	55.92 (57.12)	59.48 (75.46)	0.67
High-fat dairy	− 0.14	92.32 (140.82)	102.35 (139.52)	81.52 (140.88)	0.27
Low-fat dairy	− 0.12	138.09 (148.20)	139.52 (155.04)	135.27 (146.84)	0.27
Coffee and tea	− 0.25	750 (500)	750 (500)	637.16 (500)	0.60
Nuts	− 0.44	10.29 (13.52)	10.32 (13.79)	10.27 (13.43)	0.93
Other fats	0.31	36.70 (37.37)	32.77 (37.17)	40.51 (37.86)	**0.02**
Refined grains and starchy vegetables	0.72	559.66 (338.18)	567.29 (282.23)	543.46 (451.79)	0.82
Total DIS score	− 0.14	0.32 (1.67)	0.08 (1.48)	0.62 (1.86)	**0.002**
LIS score					
Current smoker*	0.50	17 (6%)	9 (5.3%)	8 (6.9%)	0.62^b^
Physical Activity*					
Moderately physically active	− 0.18	70 (24.6%)	43 (25.4%)	27 (23.3%)	0.051^b^
Heavily physically active	− 0.41	18 (6.3%)	11 (6.5%)	7 (6%)	
Body Mass Index (BMI)*					
Overweight BMI	0.89	116 (40.7%)	78 (46.2%)	38 (32.8%)	0.89^b^
Obese BMI	1.57	95 (33.3%)	54 (32%)	41 (35.3%)	
Total LIS score		0.89 (1.53)	0.89 (0.89)	0.89 (1.57)	0.54
DLIS score	DIS + LIS	0.99 (2.07)	0.91 (1.92)	1.26 ± 2.03	**0.04**

Unnormal and normal distributed variables were reported based on the median (interquartile range) and mean ± standard deviation. DIS: dietary inflammation score; DLIS: dietary and lifestyle inflammation score; LIS: lifestyle inflammation score.

Significant values are in bold.

*Qualitative variables were reported as frequency (percentage).

^a^*P*-value based on the Mann–Whitney test. ^b^*P*-value based on the Fisher’s Exact test.

Multivariate adjusted OR (95% CIs) for chronic migraine across DIS, LIS, and DLIS tertiles are presented in Table 4. In the unadjusted model, those in the top tertile of the DIS were significantly more likely to have chronic migraine than those in the bottom tertile [OR = 2.26; 95% CI 1.26–4.04; *P* = 0.006]. After adjustment for the effects of age, marital status, employment status, education level, migraine type, family history, pain intensity, pain duration, energy intake, BMI category, smoking, and physical activity in model 2, the association remained significant, although the odds ratio decreased slightly [OR = 2.02; 95% CI 1.06–3.82; *P* = 0.03]. In addition, the *P*-value for trend was significant in both models (P-trend model 1 = 0.005, P-trend model 2 = 0.03), meaning that participants with higher DIS were more likely to have chronic migraine.

**Table 4:** Multivariate adjusted OR (95% CIs) for CM across DIS, LIS, and DLIS score tertiles.

Score tertiles	Tertile 1	Tertile 2	Tertile 3	P for trend (r)
	OR (95% CI) *P*-value	OR (95% CI) *P*-value	OR (95% CI) *P*-value	
Multivariate adjusted OR (95% CIs) for CM across DIS score tertiles				
Model 1*	1	0.68 (0.37–1.25) 0.22	**2.26 (1.26- 4.04)** **0.006**	**0.005 (0.42)**
Model 2^#^	1	0.58 (0.30–1.12) 0.10	**2.02 (1.06–3.82)** **0.03**	**0.03 (0.34)**
Multivariate adjusted OR (95% CIs) for CM across LIS score tertiles				
Model 1*	1	**0.48 (0.26–0.87)** **0.02**	0.83 (0.46–1.51) 0.54	0.62 (-0.08)
Model 2^£^	1	**0.33 (0.15–0.69)** **0.004**	0.65 (0.29–1.45) 0.29	0.64 (-0.09)
Multivariate adjusted OR (95% CIs) for CM across DLIS score tertiles				
Model 1*	1	0.76 (0.42–1.38) 0.36	1.41 (0.79–2.50) 0.24	0.24 (0.18)
Model 2^£^	1	0.71 (0.38–1.32) 0.28	1.19 (0.63–2.26) 0.59	0.61 (0.08)

Significant values are in bold.

*P*-values based on logistic regression. DIS: dietary inflammation score; DLIS: dietary and lifestyle inflammation score; LIS: lifestyle inflammation score.

*Crude model.

^*#*^Adjusted for age, marital status, occupation status, education level, migraine type, family history, pain intensity, pain duration, energy intake, BMI category, smoking, and physical activity level.

^*£*^Adjusted for age, marital status, occupation status, education level, migraine type, family history, pain intensity, pain duration, and energy intake.

In the unadjusted model, those in the second tertile of the LIS were significantly less likely to have chronic migraine than those in the first tertile [OR = 0.48; 95% CI 0.26–0.87; *P* = 0.02]. After controlling for the effects of age, marital status, employment status, education level, migraine type, family history, pain intensity, pain duration, and energy intake in model 2, the association remained significant, although the odds ratios were slightly reduced [OR = 0.33; 95% CI 0.15–0.69; *P* = 0.004]. The *P*-value for the trend was not significant in both models. There was no significant association between DLIS tertiles and odds of chronic migraine (Table 4).

## Discussion

In this cross-sectional study, we found that higher adherence to a pro-inflammatory diet, as measured by DIS, was associated with increased odds of chronic migraine. However, no significant association was found between LIS and DLIS with increasing odds of chronic migraine. To the best of our knowledge, this is the first study to examine the association of DIS, LIS, and DLIS with migraine headaches.

Most studies examining the effects of diet on migraines have focused on the association between specific nutrients and foods with anti-inflammatory properties. For example, studies showed that anti-inflammatory foods, such as ginger^25,26^, fish oil^27^, chilis, turmeric, garlic, and onions^26^, may alleviate the neurological outcomes of migraine headache patients. Although to reduce the potential for collinearity, it might be best to focus on the inflammatory effects of a whole diet rather than on the intake of certain nutrients, food groups, or dietary components.

Our results showed that the total DIS was significantly lower in patients with episodic migraine than in those with chronic migraine. This result may be due to the significantly higher consumption of some fruits, vegetables, and legumes and the lower saturated fat intake in participants with episodic migraine. Consistent with our results, a study by Askarpour et al.^28^ showed that a healthy plant-based diet was inversely associated with a reduction in the severity, disability, and duration of headaches in women with migraine. Two cross-sectional studies in Iran suggest that a pro-inflammatory diet, as assessed by the DII score, may increase the odds of migraine headaches^14,15^; however, the results of these studies have been contradictory. Ghoreishy et al.^14^ found a positive association between the DII score and the frequency of headaches and serum inflammatory markers. In contrast, Khorsha et al.^15^ showed no association between headache duration or migraine severity and the DII score. Furthermore, a nationwide cross-sectional study using data from the National Health and Nutrition Examination Survey indicated that a higher DII score was associated with an increased risk of migraine attacks, particularly among women and young and middle-aged populations^16^. Studies have investigated the effects of various healthy dietary patterns on migraine headaches, including ketogenic diets^29^, low-glycemic index diets^30^, low-fat diets^31^, Dietary Approaches to Stop Hypertension Diets^32^, Mediterranean diets^33^, and diets with antioxidant properties^34^ or inflammatory potential^16^. Generally, migraine diet recommendations emphasize fruits, vegetables, whole grains, and lean proteins (focusing on plant-based proteins and fish) as an anti-inflammatory component, as well as a variety of foods from each food group. On the other hand, the consumption of pro-inflammatory items such as added sugars, salt, fats, and processed foods should be limited^35^.

This study found no significant association between LIS and DLIS and chronic migraine. A study of a migraine population showed that lifestyle factors such as heavy physical activity, obesity, smoking, and alcohol consumption were positively associated with migraine^36^. The results of studies on the association between smoking and migraines are conflicting. Some studies have found no effect of smoking on the risk of migraine^37,38^, whereas one cohort study followed for 7–15 years showed an increased relative risk of migraine among smokers^39^. Two meta-analyses of the available observational studies have suggested a positive association between the risk of migraine and obesity, which is likely to be mediated by gender and the frequency of migraine^40,41^. However, we did not observe a significant association between LIS and chronic migraine, which can be obtained by excluding the alcohol item from LIS calculations. Further, most of the women were inactive, overweight, or obese, and only a small percentage smoked. A possible reason for the avoidance of smoking and physical activity by patients with migraine may be that these activities are headache triggers^42^. Moreover, the extremely hot weather conditions of Ahvaz for more than half of the year should be considered a serious obstacle to being physically active in the studied population. As a result, it is reasonable to conclude that migraine does not significantly contribute to LIS in this study. More research is needed to confirm the association between a pro-inflammatory lifestyle and the likelihood of migraine.

Several studies have suggested that the association between the inflammatory potential of lifestyle and migraine may be based on possible biological mechanisms. The effect of diet and lifestyle on migraine has been studied in terms of the strength of its pro-inflammatory and anti-inflammatory effects. The DIS and LIS are indices that represent the potential inflammatory properties of diet and lifestyle based on four cytokines, including high-sensitivity C-reactive protein (hs-CRP), IL-6, IL-8, and IL-10^13^. It has been demonstrated that cytokines, by increasing permeability and interacting with cells, play an important role in the pathogenesis of inflammation and pain associated with migraine^43^. Also, an inflammatory diet and lifestyle can stimulate the trigeminal vascular system to produce a variety of pro-inflammatory neurotransmitters and neuropeptides, including the neuropeptides substance P and calcitonin gene-related peptide, which can cause neurogenic inflammation and lead to migraine^44^. In addition, inflammatory diets and unhealthy lifestyles can promote migraine by affecting intestinal microflora and the gut-brain axis^45^. For instance, consuming foods high in saturated fats, sugars, and red and processed meats could disrupt the balance of gut microorganisms and promote an inflammatory response in the intestine^46^. In addition, alcohol consumption, smoking, and obesity may induce an increase in endotoxins in the intestinal microflora, resulting in an increase in intestinal permeability and the release of pro-inflammatory cytokines^45^. This leads to intestinal inflammation, provokes the immune system, and stimulates the gut-brain axis, inducing migraine attacks^47^. Therefore, it seems that following an anti-inflammatory diet rich in fruits, vegetables, whole grains, nuts, coffee, tea, and fish may reduce migraine symptoms. Whereas sugar, refined starches, trans and saturated fatty acids with pro-inflammatory properties are associated with increased risk of migraine^33,48^.

However, it is essential to admit some limitations in the current study. The cross-sectional design of the study makes it impossible to infer the cause-and-effect relationships. Although we tried to minimize measurement errors and recall bias by applying a valid and reliable FFQ version for determining dietary intakes, some degrees of these biases are unavoidable in this study. Moreover, cultural beliefs regarding the distastefulness of women’s smoking and alcohol consumption in Iranian society can be a limiting factor in assessing the relationship between lifestyle and disease severity.

## Conclusions

According to our findings, adherence to a more pro-inflammatory diet, as revealed by a higher DIS, was positively associated with chronic migraine. However, no association was found between LIS and DLIS and migraine. To confirm the findings of the present study, further prospective studies are needed.

### Supplementary Information

Supplementary Table 1.

## Data Availability

The datasets used and/or analyzed during the current study are available from the corresponding author upon reasonable request.
