# Author Correction: Association of dietary and lifestyle inflammation score (DLIS) with chronic migraine in women: a cross-sectional study

**DOI:** 10.1038/s41598-024-70066-6

**Published:** 2024-08-15

**Authors:** Farnush Bakhshimoghaddam, Davood Shalilahmadi, Reza Mahdavi, Zeinab Nikniaz, Majid Karandish, Samaneh Hajjarzadeh

**Affiliations:** 1 Student Research Committee, Ahvaz Jundishapur University of Medical Sciences, Ahvaz, Iran; 2 Nutrition and Metabolic Diseases Research Center, Clinical Sciences Research Institute, Ahvaz Jundishapur University of Medical Sciences, Ahvaz, Iran; 3 Department of Nutrition, School of Allied Medical Sciences, Ahvaz Jundishapur University of Medical Sciences, Ahvaz, Iran; 4 School of Medicine, Ahvaz Jundishapur University of Medical Sciences, Ahvaz, Iran; 5 Nutrition Research Center, Tabriz University of Medical Sciences, Tabriz, Iran; 6 Liver and Gastrointestinal Diseases Research Center, Tabriz University of Medical Sciences, Tabriz, Iran

Correction to: *Scientific Reports* 10.1038/s41598-024-66776-6, published online 16 July 2024

The original version of this Article contained an error in the Funding section.

“This research was supported by the Student Research Committee, Ahvaz Jundishapur University of Medical Sciences under grant 330102694.”

now reads:

“This research was supported by the Student Research Committee, Ahvaz Jundishapur University of Medical Sciences under grant 02s68.”

The original Article has been corrected.

