# Supplementary material for: Association of dietary and lifestyle inflammation score (DLIS) with chronic migraine in women: a cross-sectional study
Source: Sci Rep. 2024 Jul 16;14:16406. doi: 10.1038/s41598-024-66776-6 (PMC11252145; doi:10.1038/s41598-024-66776-6)
Supplement: Supplementary file 1 — Supplementary Table 1. [file 41598_2024_66776_MOESM1_ESM.docx]

**Supplementary Table 1** Dietary and lifestyle inflammation score components.

| **Components** | **Descriptions** | **Weights** |
| --- | --- | --- |
| ***DIS components*** |  |  |
| Leafy greens and cruciferous vegetables | Broccoli, cabbage or coleslaw, cauliflower, Brussels sprouts, cooked or raw spinach, kale, mustard, or chard greens, iceberg lettuce, romaine lettuce, endive, parsley, kohlrabi, and watercress | -0.14 |
| Tomatoes | Tomatoes, tomato juice, and tomato sauce | -0.78 |
| Apples and berries | Apple, apple juice or cider, strawberries, blueberries, apple sauce, fresh blackberries, fresh raspberries, and quince | -0.65 |
| Deep yellow or orange vegetables and fruit | Cantaloupe, peach, carrots, carrot juice, persimmons, and figs | -0.57 |
| Other fruits and real fruit juices | Artichoke, Crenshaw melon, fresh coconut, fresh currants, dates, grapefruit, honeydew, apricot juice, grapefruit juice, mango juice, orange juice, other fruit juices, papaya juice, fresh pineapple or pineapple juice, prune juice, kiwi fruit, lemons, limes, mangos, nectarines, oranges, olives, papayas, tangerines, and watermelon | -0.16 |
| Other vegetables | Asparagus, beets, celery, celery juice, corn, daikon radish, eggplant, garlic, horseradish, Jerusalem artichokes, mixed vegetables, mushrooms, okra, parsnips, green or chili peppers, rutabaga, rhubarb, scallions, yellow squash, zucchini, or summer squash, turnips, and V8 juice | -0.16 |
| Legumes | Beans, fava beans, string beans, peas, peapods, alfalfa sprouts, and bean sprouts | -0.04 |
| Fish | Canned tuna fish, dark meat fish, and other fish | -0.08 |
| Poultry | Chicken with and without skin | -0.45 |
| Red and organ meats | Hamburger, liver, beef, pork, and lamb as a main dish or stew, | 0.02 |
| Processed meats | Bacon, hotdogs, and processed meat | 0.68 |
| Added sugars | Sweetened carbonated beverages, non-carbonated fruit drinks, candy bars, candy without chocolate, chocolate bars or pieces, dried fruits (apple, banana, papayas, peaches, pineapple, and mixed dried fruit), fruit cocktails, honey, jams, jellies, preserves, prunes, pudding, raisins or grapes, canned cherries, sweet pickles, and syrup | 0.56 |
| High-fat dairy | Cream, ice cream, sour cream, cream cheese, other high-fat cheese, cream sauce, sherbet or ice milk, whole milk, and yogurt | -0.14 |
| Low-fat dairy | Low-fat cottage or ricotta cheese, and skim or low-fat milk | -0.12 |
| Coffee and tea | Coffee (decaffeinated and caffeinated), and tea | -0.25 |
| Nuts | Nuts, peanut butter, seeds, and water chestnuts | -0.44 |
| Fats | Butter, gravy, margarine, and mayonnaise or other creamy dressing | 0.31 |
| Refined grains and starchy vegetables | Dark or white bread, brownies, home-baked or ready-made cakes, cold or other cooked breakfast cereal, cooked oatmeal, crackers, home-baked or ready-made cookies, doughnuts, granola bars or other granola, English muffin, bagels, rolls, muffins or biscuits, pancakes or waffles, pasta, home-baked or ready-made pastries, homemade or ready-made pie, popcorn, potatoes, French-fried potatoes, potato chips, brown rice, and yams | 0.72 |
| Other vegetables | Asparagus, beets, celery, celery juice, corn, daikon radish, eggplant, garlic, horseradish, Jerusalem artichokes, mixed vegetables, mushrooms, okra, parsnips, green or chili peppers, rutabaga, rhubarb, scallions, yellow squash, zucchini, or summer squash, turnips, and vegetable juice | -0.16 |
| Supplement score | Ranked score of supplements, including β-carotene, B-complex vitamins, calcium, copper, folic acid, iron, magnesium, selenium, zinc, and vitamins A, C, D, and E | -0.80 |
| ***LIS components*** |  |  |
| Current smoker | Currently smoked tobacco at baseline vs. did not currently smoke tobacco | 0.50 |
| Heavy drinker | Heavy (> 7 drinks [98 g]/wk) vs. non-drinker | 0.30 |
| Moderate drinker | Moderate (1 – 7 drinks [14 – 98 g]/wk) vs. non-drinker | -0.66 |
| Heavily physically active | Vigorous activity ≥ 2 times/week or moderate activity ≥ 4 times/week | -0.41 |
| Moderately physically active | Vigorous activity 1 time/week and moderate activity 1 time/week, or moderate activity 2-4 times/week | -0.18 |
| Obese BMI | Obese BMI (≥ 30 kg/m^2^) vs. normal/underweight BMI (< 25 kg/m^2^) | 1.57 |
| Overweight BMI | Overweight BMI (25 – 29.99 kg/m^2^) vs. normal/underweight BMI (< 25 kg/m^2^) | 0.89 |

BMI: body mass index; DIS: dietary inflammation score; LIS: lifestyle inflammation score
